# Supplementary material for: Barriers and Facilitators of Implementing a Healthy Lifestyle Intervention at Workplaces in South Africa
Source: Int J Environ Res Public Health. 2024 Mar 23;21(4):389. doi: 10.3390/ijerph21040389 (PMC11050208; doi:10.3390/ijerph21040389)
Supplement: Supplementary file 1 [file ijerph-21-00389-s001.zip › ijerph-2860382-supplementary.pdf]

SUPPLEMENTARY TABLE S1 : Summary of resulting themes and representative quotes from IDI and FGD participants

Table 1.1: Summary of resulting themes and representative quotes from worksite managers participating in IDIs about successful implementation of a lifestyle program

Worksite	Pre-defined domains	Themes	Summary	Representative quotes
IDI 1, 2	What would be the best times to hold lifestyle classes at your workplace?	Availability<br><br>Peak production periods	Best times will depend on employees' schedules, their mode of work and preference for lifestyle classes at the end of the day.	IDI 1, 2: "Different departments and individuals will have different work schedules."<br><br>IDI 1 "It will depend on COVID-19 level restrictions as staff work online from home during certain levels."<br><br>IDI 2: "After meetings."<br><br>IDI 2: "Preferably at the end of a workday."
IDI 3, 4			Teatime and lunch time. The day that the program would be implemented should be planned at least one week in advance. Depends on production. Very early	IDI 3: "Tea times or lunchtime."<br><br>IDI 3,4: "The best times are production driven."

Worksite	Pre-defined domains	Themes	Summary	Representative quotes
			in the morning or after hours for office workers. The afternoon is preferred since it is not too hot. During stock count towards the end of the month.	IDI 4: “It would either be very early in the morning or after hours and that only speaks to the office workers.”<br>IDI 4: “Normally afternoon is better because it is not too hot.”<br>IDI 4: “But if you do get a stock count within that time of the month, then that is a good opportunity.”
IDI 5, 6			During the day, before lunch or in the morning, or the afternoon. Communicate with production managers.	IDI 5: “During the day.”<br>IDI 5: “Before lunch, in the morning.”<br>IDI 5: “For others, it is in the afternoon.”<br>IDI 6: “Engage with production for their input.”<br>IDI 6: “Before or after the end of a shift.”
IDI 7, 8			Before or after work.	IDI 7, 8: “Before or after work time.”

Worksite	Pre-defined domains	Themes	Summary	Representative quotes
IDI 9, 10			Towards the end of the week, in the mornings.<br><br>It depends on management. Lunchtime. Last hour of the day before employees leave work.	IDI 9: "Most likely it would be towards the end of the week."<br><br>IDI 9: "If you are talking about a day. And in terms of timeline, the best time would actually be mornings."<br><br>IDI 10: "We are a very diverse company and every department works at a different pace and it all depends on the flexibility of management and how they allow for the employees to go out."<br><br>IDI 10: "Best given time would be lunchtime."<br><br>IDI 10: "Maybe the last hour of the day before they go home."
IDI 1, 2	What would be **bad times** to hold classes at your workplace?		Lifestyle classes scheduling must not encroach on department specific peak production periods.	IDI 1: "Vacation period during the festive period or during school holidays."<br><br>IDI 2: "During lunch break."

Worksite	Pre-defined domains	Themes	Summary	Representative quotes
IDI 3, 4<br><br>IDI 5, 6				IDI 2: “Not late in the afternoon as some staff would want to go home to spend time with their families.”<br><br>IDI 2: “Depends on the peak production period of different departments.”<br><br>IDI 3: “That would depend on production.”<br><br>IDI 3: “We would have to speak to the production managers.”<br><br>IDI 3, 4: “In December. We only have one week shut down.”<br><br>IDI 4: “Tea time or just after lunch.”<br><br>IDI 4: “Peak period in every month is the last week of the month.”<br><br>IDI 5, 6: “It would depend on production.”<br><br>IDI 5: “This should be communicated well in advance, maybe 6 weeks before to the production manager.”

Worksite	Pre-defined domains	Themes	Summary	Representative quotes
IDI 7,8<br><br>IDI 9, 10				IDI 6: “Shut down is around the 15th of December.”<br><br>IDI 7, 8: “Lunch breaks and tea time because it is employees’ “me” time. “<br><br>IDI 9: “And I would say lunch time. So, between 11:30 and 2:30pm is when the canteen is open and you would never find anyone or catch anyone for that time.”<br><br>IDI 9, 10: “Vacation period is generally that December period.”<br><br>IDI 9: “And then financial year end is very hectic, especially for finance.”<br><br>IDI 9,10: “The other thing is that we have our post seasonals. I know generally that is what affects the Tuesdays.”<br><br>IDI 9,10: “Peak would be beginning of the month for merchandise and end of the month for finance.”<br><br>IDI 10: “The mornings is definitely not going to work.”

Worksite	Pre-defined domains	Themes	Summary	Representative quotes
IDI 1, 2	What would be a **good location** for the lifestyle classes at your workplace?	Worksite resources	The training room and gym area is well ventilated and spacious.	IDI 1, 2: “The training room for the educational component of lifestyle classes and gym (includes a studio) for physical activity. It is well ventilated and spacious.”
IDI 3, 4			The canteen, outside the clinic area.	IDI 3: “The canteen is a great location because it is big enough space-wise.”<br><br>IDI 3,4: “We have quite a lot of space in front (outside the clinic area) to conduct the physical activity.”
IDI 5, 6			Outside the worksite building, in an open space.	IDI 5,6: “There is a good location for physical space outside on the other end. It is a very big open space.”<br><br>IDI 5: “However, we do need covering because we cannot do it in the open in case there is rain or too much heat.”
IDI 9, 10			The gym and space can be arranged for lifestyle classes.	IDI 9,10: “We do have an on-site gym.”<br><br>IDI 9: “Second to that, if you need somewhere outside then we can organise outside for a room or there's even a training room

Worksite	Pre-defined domains	Themes	Summary	Representative quotes
				downstairs. It all depends on how much space is needed."
IDI 1, 2<br><br>IDI 3, 4<br><br>IDI 5, 6<br><br>IDI 7, 8<br><br>IDI 9, 10	How do you think staff would feel about participating in online classes compared to face-to-face lifestyle education classes?	Multimodal	Both online and face to face.	IDI 1, 2: "Face-to-face and online."<br><br>IDI 3, 4: "Face-to-face and online."<br><br>IDI 5, 6: "Face-to-face and online."<br><br>IDI 7,8: "Face-to-face and online."<br><br>IDI 9, 10: "Face-to-face and online."
IDI 1, 2<br><br>IDI 3, 4	Do you think participation in lifestyle classes would be affected by gender, type of job position?	Group dynamics	Participation would not be affected by gender; however, employees may feel uncomfortable in groups with senior management.	IDI 1,2: "No problem with gender. Some employees prefer privacy and may not feel comfortable in a group."<br><br>IDI 3:" I don't think participation would be affected by gender or the manager. We have managers that are influencers."

Worksite	Pre-defined domains	Themes	Summary	Representative quotes
				IDI 3: "No, I don't think so because we do have groups whereby everyone here that is on site would meet up and there hasn't been any issues. So, I don't think there will be any issues with that."<br><br>IDI 4: "It depends on the employee's preference."<br><br>IDI 4: "From experience, I don't think that they would be comfortable if I am their manager and I am part of the group."<br><br>IDI 4: "They sometimes tend to be shy, not as free as they would be when they are with their peers."
IDI 5, 6				IDI 5: "There wouldn't be a problem as far as I know."<br><br>IDI 5: "They are comfortable because even during their breaks, they sit together"<br><br>IDI 6: "Maybe or maybe not. I don't know any conflict dynamics between them."

Worksite	Pre-defined domains	Themes	Summary	Representative quotes
IDI 7, 8<br><br>IDI 9, 10				IDI 6: "You will find employees that always feel inferior or get intimidated if there is a manger."<br><br>IDI 7, 8: "No, they would be fine."<br><br>IDI 9, 10: "No. It shouldn't be affected but like I've been saying, if people have the time, then everyone would be fine."
IDI 1, 2	What topics about healthy eating or physical activity do you think will most interest your employees?	Healthy lifestyle	Healthy eating and healthy lifestyle including practical tips. Topics of interest should be the same for all employees.	IDI 1: "Weight control, maintenance of cholesterol, high blood pressure, diabetes, healthy eating, healthy lifestyle and lifestyle modification."<br><br>IDI 2: "Eating plans for employees. Planned meals by a dietician delivered to employees for lunch (which was practiced pre-COVID-19."<br><br>IDI 2: "Videos on preparing healthy and easy meals can be sent to employees."<br><br>IDI2: "Employees eat with their eyes, healthy meals should be appealing, convenient and simple to prepare."<br><br>IDI 2: "Demonstrate different methods and techniques of using various equipment such

Worksite	Pre-defined domains	Themes	Summary	Representative quotes
IDI 3, 4				as preparing a meal in the air fryer versus oven.”<br><br>IDI 2: “Topics will be the same for all employees. The workplace has a diverse culture and caters for everyone.”<br><br>IDI 3: “For females, it is alwretays weight loss and maintenance and with males, it is mostly about diabetes.”<br><br>IDI 3: “Others just have a general interest in being healthy.”<br><br>IDI 3,4: “People really do want to know the type of diets and what's good to eat and what's not good to eat.”<br><br>IDI 3: “I've heard recently people speaking about blood groups and what types of food that you need to eat depending on your blood type.”<br><br>IDI 4: “Wellness”<br><br>IDI 3,4: “The topics should be the same for all employees.”

Worksite	Pre-defined domains	Themes	Summary	Representative quotes
IDI 5, 6				IDI 5: “They are interested in aerobics.”<br><br>IDI 5: “Nutrition, nothing in particular.”<br><br>IDI 6: “Obesity, portion sizes, healthy food options, create awareness.”
IDI 7, 8				IDI 7,8: “Balanced diet, healthy meals.”<br><br>IDI 7: “Healthy foods that I can eat to avoid taking medication.”<br><br>IDI 8: “Depression and signs on how to recognise depression to actually help somebody, try and rescue them.”
IDI 9, 10				IDI 9: “I would think the nutritional side behind things. So, when a person is choosing the food, what are they actually choosing.”<br><br>IDI 9: “The food categories, your nutrition elements as to like how many calories are in a meal and the amount of exercise needed to burn what you consumed.”

Worksite	Pre-defined domains	Themes	Summary	Representative quotes
				IDI 9: "Health tips like I know turmeric helps with headaches."<br><br>IDI 9: "If you have interesting facts, our employees would love that."<br><br>IDI 9, 10: "In terms of lifestyle, I would also say tips for easy exercises that employees could do at their desk or walk around the worksite."<br><br>IDI 10: "A balanced diet. We could talk about a balanced lifestyle."<br><br>IDI 9, 10: "One topic for everyone."
IDI 1, 2	What would encourage your employees to participate in the lifestyle classes and why?	Incentivisation, worksite support and engaging lessons	Reward employees, interesting educational tools, support from management and peers, and fun lessons.	IDI 1: "Trying to reward them or create attractive leaflets."<br><br>IDI 1,2: "Managers are unaware of employees' medical conditions, but they may support them; however, it is an individual's choice."<br><br>IDI 1: "Type of classes and how they are conducted."<br><br>IDI 1: "Support from co-workers."

Worksite	Pre-defined domains	Themes	Summary	Representative quotes
				IDI 2: “The main aim is to create awareness to encourage employees to participate.”
IDI 3, 4				IDI 3,4: “Gifts, rewards and incentives.”<br><br>IDI 3,4: “The company is very health driven so managers will definitely buy in.”<br><br>IDI 4: “If you want to encourage people, show them the benefits.”<br><br>IDI 4: “I am thinking to give them free time as a form of support.”<br><br>IDI 4: “Support is always important whether it comes from friends or family members.”
IDI 5, 6				IDI 5, 6: “Reward or an incentive so no matter how small the incentive is, they will come.”<br><br>IDI 5: “Support from the managers in terms of how do we get these people at the same time.”<br><br>IDI 6: “Make it fun, have a team leader, engage with staff, know their interests, have

Worksite	Pre-defined domains	Themes	Summary	Representative quotes
				competitions, offer support and motivate staff, create health awareness.”
IDI 7, 8				IDI 7: “The motivation and what is in it for me as an individual.”<br><br>IDI 7: “Communication.”<br><br>IDI 8: “Self-driven, the need for change as an individual to be healthier so you can look good and walk around feeling better.”<br><br>IDI 8: “Colleagues, because if we are on the same journey then we can discuss how they are coping, the challenges and how they are overcoming it.”
IDI 9, 10				IDI 9,10: “Rewards and incentives.”<br><br>IDI 9,10: “Make employees aware of the benefits of participating in the program.”<br><br>IDI 9: “And third, definitely a big one, is changes to the canteen. Introducing a variety of meal options.”<br><br>IDI 9: “Our guys love competitions.”

Worksite	Pre-defined domains	Themes	Summary	Representative quotes
				IDI 9: “If you have support from all angles, you are going to fly high.”<br><br>IDI 10: “Time for me… if I get authorization from my manager or my line manager and she is completely fine with it then only I would not mind doing it.”<br><br>IDI 9,10: “Managers won't really have a problem, but depending on how busy they are.”
IDI 1, 2<br><br>IDI 3, 4	What might be the challenges for your employees to participate in the health classes?	Barriers to participation	Production deadlines, fear of maintaining confidentiality, feeling uncomfortable in participating in a group, connectivity challenges for online classes and support from managers.	IDI 1, 2: “Time, maybe due to workload, but that'll depend on when it's conducted.”<br><br>IDI 1: “Exposure, the concern of maintaining confidentiality, therefore not wanting to share their personal experiences.”<br><br>IDI 3: “Production issues.”<br><br>IDI 3: “Some of them will feel embarrassed to exercise in front of other people.”

Worksite	Pre-defined domains	Themes	Summary	Representative quotes
				IDI 3: “Others won’t be comfortable changing into their comfortable gym clothes in front of other people.”<br><br>IDI 4: “Time.”<br><br>IDI 4: “Online classes might have connectivity issues.”
IDI 5, 6				IDI 5: “This is a food factory so cleanliness and hygiene is very important.”<br><br>IDI 5: “They can’t walk with their overalls outside so they need to change into their personal/gym clothes.”<br><br>IDI 5: “They should do the physical activity, come back, take a quick shower or refresh themselves and go back and change into their white overalls.”<br><br>IDI 5,6: “Time.”<br><br>IDI 6: “Support from managers.”

Worksite	Pre-defined domains	Themes	Summary	Representative quotes
IDI 7, 8<br><br>IDI 9, 10				IDI 7: “Change within yourself.”<br><br>IDI 8: “Time.”<br><br>IDI 9,10: “Time management.”<br><br>IDI 9: “If somebody is in the program and they are not seeing progress, then they might want to step off.”<br><br>IDI 10: “Management.”
IDI 1, 2	What else should we consider when designing the healthy lifestyle program for your workplace and employees?	Barriers to participation	The lifestyle program should be open to all staff, should include healthy eating, take into account cultural practises, classes should be repeated and there must be open communication.	IDI 1: “Include all employees in the wellness program.”<br><br>IDI 1: “Avoid stigmatism by using a general term to name the program, for example, wellness program.”<br><br>IDI 2: “Ensure the interventions are implemented within the canteen environment.”<br><br>IDI 2: “Employees bring homemade meals for lunch; therefore, they should be made aware that their portion size should be similar to meals served at the canteen.”

Worksite	Pre-defined domains	Themes	Summary	Representative quotes
IDI 3, 4				IDI 3: “I think we should consider likes and dislikes of employees.”<br><br>IDI 3: “And a variety of healthy menu options.”<br><br>IDI 3: “Take employees’ cultural backgrounds into consideration.”<br><br>IDI 4: “I think you should open it up to even more people, not only the people who you think are at risk because you will find that there are people who feel that they also need it.”
IDI 5, 6				IDI 5: “In terms of food, try to accommodate every person, everybody, all cultures.”<br><br>IDI 6: “Mental health, social eating.”
IDI 7,8				IDI 7: “The cultural background of employees.”
IDI 9, 10				IDI 9: “Main thing would be the timelines. Do multiple classes instead of just one class a week to accommodate all employees.”<br><br>IDI 9: “Speak to people that are joining to see what their preferences are.”

Worksite	Pre-defined domains	Themes	Summary	Representative quotes
				IDI 9: "Keep the lines of communication going."<br><br>IDI 9: "Find out which medium of communication the participant would prefer (calls, emails, Teams, etc.)."<br><br>IDI 10: "Apart from the nutritional food and physical activity, I think group sessions. it could be something totally diverse and how people deal with their pressures and stressors every single day. So, I would say more of a group session of motivation."

Table 1.2: Summary of resulting themes and representative quotes employees participating in FGDs about healthy eating

Pre-defined domains	Themes	Summary	Representative quotes
What do you understand by healthy foods and beverages?	Meaning: Nutrient-dense foods and water	Foods that are that nutrient-dense, fruit and vegetables, foods cooked using healthy cooking methods and consumed in correct portion sizes. Beverages should include mainly water and contain reduced sugar.	KP3, KP1, KP4: "Healthy food and beverages is basically eating food that have all the nutrients and vitamins that the body needs."<br>KP3, KP1: "Vegetables and fruit - An example of vegetable is green vegetables (spinach) and the benefit is that it prevents constipation."<br>KP3, KP2: "Orange for vitamin C."<br>KP2: "Vegetables help the body to function."<br>KP4: "Carrots, broccoli and other vegetables that are sources of vitamins to ensure that you have a balanced diet."<br>KP4: "You can have pumpkin with the skin on which helps with roughage - makes your stomach more regular"<br>KP4: "A healthy diet should contain all the elements which makes it a balanced diet"<br>KP3: "Meat for protein - beef"<br>KP4, KP1: "White meat such as chicken with the skin off, fish grilled, boiled, baked without introducing extra oil that will raise your cholesterol."<br>KP2: "Boiled food."

Pre-defined domains	Themes	Summary	Representative quotes
			KP4: “Also the way you prepare food. You find that the meat is healthy but the manner in which you prepare it makes it very unhealthy for you. Healthy methods are baking, steaming or grilling, make sure you do not put in a lot of salt because it results in hypertension and oedema.”<br>KP4: “Healthy beverages are something that does not contain a lot of sugar because it would change into fat and lead into chronic issues.”<br>KP4: “Water would be the best beverage to drink, it is good for your body and cleans out the toxins.”<br>KP4: “There are different categories in terms of which you can have your starches. You can have your pap. Instead of having pap, you can have your low GI bread, instead of white rice, you can have brown rice or basmati rice”<br>KP4: “The portion size is very important.”<br><br>MP1: **“**Beverages have to be healthy with no alcohol.”

Pre-defined domains	Themes	Summary	Representative quotes
			MP1: "Unprocessed foods, more fruits and vegetables, less starch such as potatoes and rice, less foods that are processed."<br><br>MP2: "Healthy food is like the opposite of fatty, sugary foods (lasagne, pies, sugar drinks, Coke and Sprite, fast foods like MacDonald's, processed foods)."<br><br>MP2: "Brown rice, your veggies like broccoli, cauliflower, like fresh vegetables."<br><br>MP2: "It's usually unprocessed foods."<br><br>MP2: "Healthy organic foods"<br><br>MP3: "To eat vegetables with your meat."<br><br>MP3: "You can eat starch (such as potatoes) but not a lot of starch and also not too much of oil."<br><br>MP4:" Water, boiled meals such as boiled vegetables."<br><br>LP1, LP2, LP4: "Water is the best".

Pre-defined domains	Themes	Summary	Representative quotes
			LP2: “Protein that is fresh and lean cut such as a piece of steak with salad and vegetables or chicken salad to have a mixed meal”.<br><br>LP5: “I have a slightly different perspective that everything is good or bad for you depending on the quantity consumed.”<br><br>LP4: “Homemade meals and 100% fruit juices.”<br><br>IP6: “Water.”<br><br>IP6: “I would steam my broccoli and cauliflower and make sure that it has a crunch.”<br><br>IP3: “Food should be eaten in moderation as opposed to excessive eating because you can have like the junk food but it has to be in moderation.”<br><br>IP2, IP6, IP1: “More of a balanced diet, eating fruits more often, having boiled or steamed food rather than fried or foods that are cooked with a lot of oil.”

Pre-defined domains	Themes	Summary	Representative quotes
			IP1: “Healthy eating is something that will be good for your body and give you all sorts of vitamins as well.”<br><br>RP1, RP4: “Balanced diet. There has to be a balance with all the food groups in your diet.”<br><br>RP4: “Knowing how much of calories we are putting onto our plate.”<br><br>RP1: “It’s not that you can’t have sugar but you should have a limited amount of sugar.”<br><br>RP1: “Chicken fillet prepared in an air fryer, broccoli and cauliflower in an air fryer or steamed in the microwave.”<br><br>RP2: “Balance in terms of what you are putting into your body versus what you should put in your body.”<br><br>RP2, RP3. RP1: “Foods prepared in an air fryer”<br><br>RP3: “Reduce fizzy drinks with sugar completely.”

Pre-defined domains	Themes	Summary	Representative quotes
			RP3: "Water is the best."<br><br>RP3: "Preparing food with olive oil."
What do you understand by unhealthy foods and beverages?	Meaning: Nutrient-poor foods and energy-dense beverages.	Fast food, and processed food. Foods high in fat, salt and sugar and energy consumed in excessive amounts.<br><br>Sugar-sweetened beverages and alcohol.	KP4: "Foods like McDonalds, French fries and burgers."<br>KP4: "Drinks like cold drinks."<br>KP3: "Spicy food and energy drinks."<br>KP1: "Alcohol."<br><br>MP1: "Junk food, chocolates, cold drinks, fruit juices have too much sugar so it is not healthy."<br><br>MP1: "Biscuits, sweets, those kinds of things."<br><br>MP2: "It is foods that are likely to make you gain weight, not good for you, like too much sugar causes diabetes."<br><br>MP2: "Eating fast food."<br><br>MP3: "Unhealthy food leads to diseases like high blood pressure, diabetes and obesity."

Pre-defined domains	Themes	Summary	Representative quotes
			MP3: “Eating a lot of starch such as potatoes.”<br><br>MP4:” Eating chips, cookies, too much sugar, too much salt, red meat - Shisanyama.”<br><br>LP1. LP2: “Foods high in starch.”<br><br>LP1, LP3: “Excessive consumption of food.”<br><br>LP1, LP4: “Drinking Coke and sugar sweet and beverages. Juices are high in sugar.”<br><br>LP4: “Take-aways and skipping of meals.”<br><br>LP4, LP3: “Excessive alcohol intake.”<br><br>LP2: “Burgers, pizzas, foods that are greasy and oily, less protein, less greens in it.”<br><br>LP2:” We are trying to lose weight, but Mr Delivery was our best friend when we were working at the office during COVID-19 because the canteen was closed.”

Pre-defined domains	Themes	Summary	Representative quotes
			LP2: “The employees at the factory sites prefer meals with a large portion of starch, for example, phuthu, pap, rice, pasta and samp as it is their comfort foods.”<br><br>LP2:” Fizzy drinks are fast sellers, but water is our way forward for everyone.”<br><br>IP6, IP3, IP1: “Junk food, fast foods so like McDonald’s.”<br><br>IP3, IP1: “Something with a lot of oil in it…processed foods.”<br><br>IP2, IP4: “Fried chips, vetkoeks.”<br><br>IP1, IP4: “Burgers, you can have a very nice fresh fish but it’s how it is prepared, if it is processed then it becomes unhealthy.”<br><br>IP4: “Foods with excessive fat and excessive sweetness. If there’s no balance, then it is unhealthy.”<br><br>RP4: “Sugary foods.”<br><br>RP2, RP1: “Overindulgence, unbalanced diets.”

Pre-defined domains	Themes	Summary	Representative quotes
			RP2: “Fast foods such as KFC.”<br><br>RP2: “Also how you prepare a meal depends on the healthiness of it.”<br><br>RP4: “Large portion sizes.”<br><br>RP4: “Sugary and fatty foods, eating these would cause heart disease at a very young age.”
What are the factors that determine your food and beverage choices?	Food environment<br><br><br>Healthy longevity	Convenience, cost of meals, taste, environment, promotions, family influence, culture, stress/ mood.	KP4: “Time. if you don’t have time to carry a lunchbox to work then you would go to the canteen because you are hungry and need something to eat.”<br>KP4: “The prices, healthier meals are much more expensive than the unhealthy meals which is a difficult decision to make and I would choose the lower priced meal.”<br>KP1: “The surrounding area that you are in.”<br>KP1, KP2, KP3, KP4: Personally, yes, if there was a coupon or discount then we would purchase healthy meal options.”<br>KP3: “Unhealthy foods taste better than healthy foods. If I had to choose boiled meat compared to the braaied meat, the

Pre-defined domains	Themes	Summary	Representative quotes
			braaied meat would taste better with all the spices.”<br><br>MP1: “Taste.”<br><br>MP2: “Finance and sometimes convenience but most of the time will be finance.”<br><br>MP3: “Because I need to stay healthy all the time and to avoid getting any diseases. Some people die from obesity because they do not manage their diets.”<br><br>MP4:” My wife prepares the meals.”<br><br>MP4:” The children influence meal choices.”<br><br>LP3: “Your medical conditions would determine what is good or bad for you.”<br><br>LP1, LP4: “Type of environment, family and cultural factors.”<br><br>LP5: “I’m a Hindu so I don’t eat beef or pork, I eat vegetables three times a week.”

Pre-defined domains	Themes	Summary	Representative quotes
			LP1: “When I’m staying by myself, its more convenient because I’m always on the go.”<br><br>LP1: “It’s my time that predicts what I'm going to eat or how I plan my menu for the week ahead.”<br><br>LP5, LP4, LP3: “I agree with P1. It’s your mood, location, how much money you have, whether you are at family functions or a friend’s home, calorie count determines the amount that I consume.”<br><br>LP4: “Also determinants like hunger and appetite.”<br><br>IP6: “Mood.”<br><br>IP2: “It would depend if I’m consuming alcohol.”<br><br>IP3: “I think for me it is what my body can tolerate and what it can’t.”<br><br>IP4: “I think it would be the same as P6 said, my mood, cravings you know as well as celebrations.”

Pre-defined domains	Themes	Summary	Representative quotes
			IP3: "Stress levels."<br><br>IP1: "For me, it depends where I am."<br><br>IP1: "And also what is available at that point in time."<br><br>IP2, IP4, IP5: "Cost."<br><br>IP4: "When I lived with my parents, the whole household was eating what was prepared. I was vegan for 6 months. It was difficult, not because it was time consuming but when they went grocery shopping, they didn't account for certain things."<br><br>RP3, RP2, RP1: "Convenience."<br><br>RP4: "Time."<br><br>RP2: "Affordability."<br><br>RP1: "What gets advertised out there, for example, people are always at KFC and McDonald's but you'd never see a deal at Woolworths, for your vegetables or healthy meals."

Pre-defined domains	Themes	Summary	Representative quotes
			RP4: “Wanting to see a change within yourself.”<br><br>RP4: “The meals are already prepared when I get home so I eat whatever is there.”<br><br>RP4, RP2, RP3: “Stress, boredom, mood.”<br><br>RP1: “Taste and satisfaction.”
What facilitates you to choose healthy food and beverages?	Mindful food choices<br><br>Enabled healthy food environment	Mind-set of an individual, health conditions, weight control, affordability,<br><br>The individual preparing the meal at home, personal preferences, taste, time, convenience, eye-catching meals, peers/the people you surround yourself with, blood type, the need to be healthy, healthy food enables you to concentrate better, availability, accessibility.	KP4: “My mind-set.”<br>KP4: “If you have a condition that restricts you from eating unhealthy food.”<br><br>MP1: **“**My weight, I was not this fat but after giving birth…”<br><br>MP2: “If it is affordable then I would eat it because healthy food tastes good.”<br><br>MP4: “My wife prepares my meals; I don’t like spicy food.”

Pre-defined domains	Themes	Summary	Representative quotes
			LP3, LP5: “It’s taste because I’m a very picky and fussy eater and stress levels”.<br><br>LP5: “The time available to have lunch determines the type of meal purchased from the canteen. For example, if the salad queues are long, it is easier to purchase a pie.”<br><br>LP5: “Yes, knowledge is power. When I found out that I’m pre-diabetic, I’ve been doing research.”<br><br>LP1, LP4: “Convenience and what’s attractive to the eye.”<br><br>IP4: “My family has medical issues that I’m trying to avoid in the future.”<br><br>IP2: “I want to remain healthy all the time.”<br><br>IP1: “When you eat healthy food, you are not too full, you feel light and you are still able to concentrate, you got good energy levels whereas when you eat junk, you want to sleep, you are not productive anymore. It is tasty but afterwards, there are no benefits.”

Pre-defined domains	Themes	Summary	Representative quotes
			RP3, RP2, RP1: “Your company/peer group”<br><br>RP2, RP4: “Availability and having access to healthy meals.”
What prevents you from choosing healthy food and beverages?	Barriers to healthy food access	Availability, affordability, convenience, mood, skill to prepare meals, limited variety of healthy meals, poor sensory appeal of healthy meals.	KP4: “Availability.”<br>KP3: “Affordability.”<br><br>MP2: “Affordable ingredients, if I can’t cook it myself. I usually make rice and curry because that is cheap.”<br><br>MP3: “Where you are staying because I’m staying alone. I do whatever I feel that is healthy for me. Sometimes, I have cereal (cornflakes) in the morning and the next meal is maybe at 6pm because I am busy which is unhealthy for me.”<br><br>MP4: “Time to prepare meals.”<br><br>IP3. IP2: “Cost”.<br><br>IP3: “I work overtime so meal preparation is so time consuming whereas with the

Pre-defined domains	Themes	Summary	Representative quotes
			quick junk meal, you can just do something that is easy."<br><br>RP2: "Same menu repeated, there's no variety."<br><br>RP2: "Taste and texture."<br><br>RP1: "Healthy foods always look the same no matter which place you're eating at."
What would be the best way to organise lifestyle classes at your worksite (location, time of day, composition of groups)?	Scheduling and mode	Multimodal lifestyle classes with proper scheduling from managers; the best time would be during the day around shift change; staff on leave must be accommodated; size of class will depend on training venue size; communicate lesson time in advance.	KP3: "It has to be onsite."<br>KP4: "Face-to-face under supervision instead of online because people cheat."<br>KP3: "Get time allocated from production so that I know I have this one specific hour that I have apart from our lunch breaks, after all we still need our lunch breaks."<br>KP3: "The number of people that we have in a group does not matter but it would depend on the space that we have."<br>KP2: "Management should be included."<br>KP2: "During the break because we cannot come in the morning, get tired and work."<br>KP1: "Take into consideration public transport."

Pre-defined domains	Themes	Summary	Representative quotes
			KP3: "It should be during the day."<br><br>MP1: "The admin staff should do it on their own because admin is busy Mondays to Fridays."<br><br>MP1: "It would depend on the shifts because usually the employees working the 2pm to 10pm shift is always here early and can do something but for the employees that are here at 6am and leave at 2pm it would be a challenge."<br><br>MP1: "Morning shift is always hectic, because they are always covering up for lost time at night."<br><br>MP1: "I prefer face-to-face, online is just over-rated."<br><br>MP2: "I think you are most likely to get the employees during work time so you know they are already at work and it is easier to access them."<br><br>MP2: "I would say in the beginning of the week on a Monday or Tuesday because everyone is still fresh. If you get someone

Pre-defined domains	Themes	Summary	Representative quotes
			to come in on a Friday, they would be less likely to do it.”<br><br>MP2: “Also in the mornings, around 10am is a good time to start or between 8-10am are good hours, 12-2pm is going to be hard.”<br><br>MP2: “Yes, I’d be comfortable in a group, because we are all in it for the same goal.”<br><br>MP3: “If it is online, I would have to be on my phone so I can take my lunch time to do it.”<br><br>MP3: “Anytime, we can plan time aside when I am working on either one of my three shifts.”<br><br>MP3: “Anyone in a group is fine.”<br><br>LP5: “No, I wouldn’t be comfortable in a group. I’m a very reserved person. To me, my condition is personal, that I need to self-correct. I wouldn’t want the spotlight on me."<br><br>LP5: “I’m not too happy with doing physical activity at work due to sweating

Pre-defined domains	Themes	Summary	Representative quotes
			and having to go back to work. I do go to the gym”.<br><br>LP2: “Employees prefer one-on-one sessions as they have a private lifestyle".<br><br>LP2: “Throughout the year so you take your leave as you want”.<br><br>LP2: “There is a structured one-hour lunch break, but employees sometimes work through the breaks.”<br><br>LP2: “Lifestyle classes should be scheduled according to the employees’ availability.”<br><br>LP3: “From the morning until 2pm is my busiest time of the day and I would not be available. I’m quite happy to participate in any physical activity that you may plan after 2pm”.<br><br>IP1, IP2, IP3, IP4, IP5, IP6: “Onsite, where everyone is.”<br>IP3, IP1, IP4, IP5: “Before lunch time, not during the break.”<br>IP6: “In the morning.”<br>IP1: “Yes, the mix is fine. It is perfect.”

Pre-defined domains	Themes	Summary	Representative quotes
			RP1: "Online classes would work for the company because people have less time or are travelling."<br><br>RP2: "Depends on an individual's lifestyle. For me, I'd prefer face-to-face."<br><br>RP2, RP1: "Location would definitely be the gym."<br><br>RP2, RP1: "Groups would work but not too big groups because people would tend to lose focus"<br><br>RP1: "Five to ten people in a group to get motivation from each other as well."<br><br>RP1, RP2, RP3: "I don't mind different employees in a group but it would depend on each employee if they are intimidated."<br><br>RP3: "I don't mind because you would get to meet people from other departments and you might just gel with them, it would be diverse."<br><br>RP2: "Provide notices/communicate in advance."

Pre-defined domains	Themes	Summary	Representative quotes
What would facilitate your participation in lifestyle classes?	Healthy longevity<br><br>Scheduling of lifestyle classes	Individuals drive and mind-set, support and motivation, weight control,<br><br>Achieving your goals, the need to live healthy and reduce the risk of developing chronic diseases, health status, the outcome and benefits,<br><br>The need for change, sharing of information with family members and multiple classes to accommodate all employees.	KP4, KP3: “My drive and mind-set.”<br>KP2: “If there is support and motivation through the process.”<br><br>MP1: “I want to lose weight. Losing weight is my number one goal.”<br><br>MP1: “Healthy living. I don’t have any chronic diseases yet, but I don’t want them in the near future.”<br><br>MP2: “To achieve the goals, like I know what I want to achieve.”<br><br>MP3: “It’s going to be good for my health.”<br><br>IP4: “Your health.”<br>IP6, IP3: “The outcome and benefits.”<br>IP5: “And also, you see the need to change.”<br>IP5: “Whatever you learn here, you can try and take it home.”

Pre-defined domains	Themes	Summary	Representative quotes
			RP2: “Communicate in advance so time can be allocated for employees to participate.”<br><br>RP2: “Multiple classes to accommodate different employee schedules.”<br><br>RP4: “Support from management.”
What would prevent your participation in lifestyle classes?	Time limitations<br><br>Self-efficacy	Time limitations due to job responsibilities and self-efficacy	KP4: “Time.”<br>KP3: “The feeling of being tired and not wanting to go back to do work.”<br>KP2: “Laziness.”<br><br>MP2: “Unachievable goals, if it is something that I don’t like or see that it is not benefiting me in any way then I will start to feel detached from it.”<br><br>MP3: “As long as it’s not going to affect my job and my time because we are working.”<br><br>IP5, IP4: “Time”.<br>IP3: “My mood”.<br>IP2: “It would be laziness”.<br>IP2: “Maybe job challenges”.<br>IP6: “It’s the drive for me.”

Pre-defined domains	Themes	Summary	Representative quotes
			LP5, LP2: “Meetings”.<br><br>RP3, RP4: “Time”.<br><br>RP1: “If people have to shower and freshen up after exercising then it would be a challenge.”
In your opinion, do you consume the same, less or more food and beverage when you meet with family and friends?	Social eating	More food and beverages are consumed with friends and family, at home and at work.	KP2: “When we have friends, we are eating more junk food”.<br>KP2, KP3: “More food consumed at work at the canteen than at home because we can ask them to add more starch, not protein.”<br>KP3: “It would depend on where we are”<br>KP4: “I think it would depend”.<br>KP4: “Socially, it would depend. If it is with extended family then there are different varieties and you would consume more but if you with immediate family, I don’t think that it would change.”<br>KP1: “I eat more at home; at work there are small portions, or I do not eat at all when I am at work.”

Pre-defined domains	Themes	Summary	Representative quotes
			MP1: "Family and friends, yes more because I hardly eat a lot on my own."<br><br>MP2: "Definitely more."<br><br>MP2: "At work, I am less likely to eat more than when I am at home. At work it is difficult because we only have a few breaks and I usually only eat during one break.<br><br>Recently, I have been eating healthier but usually foods that are cheaper and convenient such as chicken pasta."<br><br>MP3: "More, I eat a lot when I am with my family."<br><br>MP3: "When I'm with my friends depends where we going or when I am going to a braai and there's a lot of meat, yes, but when I'm by myself I do not eat a lot."<br><br>MP4:" Yes, more food is prepared to accommodate family and friends that are over. More fancy meals are prepared."

Pre-defined domains	Themes	Summary	Representative quotes
			IP2: “More especially, I consume healthy food at work but if I’m at home I’m not this healthy.”<br><br>IP1, IP2, IP3, IP4, IP5, IP6: “More.”<br>IP5: “Way more.”<br>IP6: “With the environment and vibes, you end up eating more. There are different types of food.”<br>IP6, IP1, IP5: “Unhealthy foods, meat, salads”<br>IP3: “And we don’t eat a lot of salad, more meat.”<br>IP5: “Fizzy drinks, alcohol.”<br>IP1, IP2, IP4, IP5: “No, this is not the same whilst at work.”<br>IP3: “It is different, unless there’s a function or event where we are eating and not working.”<br>IP6: “We don’t eat with colleagues due to social distancing so it’s not more. If we are sitting with friends or eating alone at work, it would be the same quantity.”<br><br>RP3, RP4: “More”.<br><br>RP1: “Definitely more because eating is part of the social aspect. There’s snacks,

Pre-defined domains	Themes	Summary	Representative quotes
			mains and then dessert because maybe someone has a sweet tooth."<br><br>RP2: "More especially at family gatherings."<br><br>RP3: "There is a variety of food options available."<br><br>RP2: "It also depends on how many people there are."
Pre- and post COVID-19, did you notice any changes with your food and beverage consumption?	Mixed eating patterns induced through emergency situations	Food and beverage consumption varied remaining the same for some whilst changed towards healthy eating choices.	KP4: "The same for me since I still had to work and was not at home."<br><br>KP4: "I consumed more fruits and vegetables for the extra vitamins and nutrients that I need."<br><br>KP3: "Yes, I was consuming more food during COVID-19 but they were unhealthy due to what was available because we were limited and remember some shops were closed. People were not working so the availability of food."<br><br>KP2: "I consumed more boiled veggies because we were scared about the virus."

Pre-defined domains	Themes	Summary	Representative quotes
			MP1: **"**Before COVID-19 I don't think anyone actually cared about what they ate, after COVID-19 happened we all tried this healthy living thing because we are going to die from COVID-19."<br><br>MP2: "Yes, I prefer drinking sparkling water now and coffee (black with sugar) so that I don't drink cold drink."<br><br>MP3: "I didn't see any changes for me, because I didn't get sick. I'm just eating as normal as I did before."<br><br>LP5: **"**Pre COVID-19, my medical statistics were good. Whilst working from home during COVID-19, I am now pre-diabetic and have high cholesterol, it was not the type of food consumed but the amount consumed."<br><br>LP2, LP3: "Prior to COVID-19, we were dishing and because of different people serving, there was an inconsistency in portion control. Pre-packed meals assist in better portion control; therefore, employees are finding that the meals are insufficient. When meals were dished up,

Pre-defined domains	Themes	Summary	Representative quotes
			employees would ask for a larger portion for the same price."<br><br>IP1, IP2, IP3, IP4, IP5, IP6: "Yes."<br>IP6: "Especially the beginning of COVID-19."<br>IP5: "I was so healthy. During COVID-19, I gained so much weight and I was just eating junk all the way."<br>IP5: "I was eating anything because remember, you are just confined in one space. You used to work and watch television whilst being busy with chocolates, hot chips, you are buying takeaways all the time because you don't want to cook."<br>IP1: "I could eat more. It would be frequent because I felt I could eat all the time. Like I will go and work and then look for something like a chocolate."<br>IP6: "Or make food because everybody was a chef that time. I was baking, like scones… cooking, because you have the time you know."<br>IP4: "I've gained a lot of weight because of eating and drinking so much. It's my weakness. I had to finish up all the stock that was at home. I was just travelling from work to home, home to work."

Pre-defined domains	Themes	Summary	Representative quotes
			IP3: “It was the same. There was no need for me, it was like the same things because I’m in the house, it’s the same food we buy on a weekly basis.”<br>IP6: “It got worse because of the anxiety I was having. I never lived under pandemic conditions, because of the different levels, I buy everything and have it. When they say takeaways or restaurants are opened, I would go and buy and eat. So, each time I get a chance, I would just eat everything.”<br><br>IP2: “During COVID-19, I was consuming more and post COVID-19, I went back to normal because I was panicking of my health.”<br><br>RP3: “Yes because everything was closed during lockdown, people were buying lots of groceries and you had time to prepare.”<br><br>RP3, RP1: “Overindulging during COVID-19 but post COVID-19, we started getting back to the routine.”

Pre-defined domains	Themes	Summary	Representative quotes
			RP2: “Yes, I had COVID-19 so I lost my taste and smell and that put me off from eating.”<br><br>RP4: “Not many facilities were opened to us. We had no access to restaurants, only convenient stores, gyms were also closed. Everyone switched off and turned to food. I lost my appetite when I had COVID-19.”<br><br>RP3: “I was pregnant when I had COVID-19 and I had to eat to sustain myself.”
